# Investigation of Measurement Cycle Reproducibility and Dynamic Resistance-Switching Capability in Reduced Graphene Oxide

**DOI:** 10.1021/acsomega.5c10458

**Published:** 2026-02-24

**Authors:** Ricardo Barreto da Silva, Artur Harres de Oliveira, Josué Neroti Rigue, Carolina Ferreira de Matos Jauris, Fernando César Comparsi De Castro, Luís Henrique Schneider, Candice Müller

**Affiliations:** † Departamento de Física, 28118 UFSM, Santa Maria 97105-900, Rio Grande do Sul, Brazil; ‡ Departamento de Química, UFSM, Santa Maria 97105-900, Rio Grande do Sul, Brazil; § Departamento de Eletrônica e Computação, UFSM, Santa Maria 97105-900, Rio Grande do Sul, Brazil

## Abstract

Reduced graphene oxide (rGO) has been employed as a variable resistor in the development of RF and microwave devices, enabling electronic tuning of these systems. This paper investigates the experimental behavior of four different graphene samples in a microstrip with a gap attenuator structure. Beyond conventional DC resistance analysis, we assess the reproducibility of resistance across multiple measurement cycles and examine the dynamic resistance-switching capability and memory effect under current stepping. All samples exhibited resistance variation under the applied DC bias. However, this variation was not reproduced in subsequent measurement cycles, resulting in a reduced dynamic variation of the graphene resistance. Notably, the resistance variation decreased significantly from the first to subsequent cycles. Current step measurements revealed that rGO exhibits a memory effect, leading to resistances lower than the previous ones for a current of 1 mA, after being submitted to currents higher than 10 mA. Radio frequency measurements demonstrated that transmittance can be tuned via DC bias, with tunability strongly dependent on individual sample characteristics. Such behavior compromises the graphene’s dynamic switching capability. Most existing studies highlight the tunability of graphene-based RF devices; our findings reveal, for the first time, that reduced graphene oxide exhibits cycle-to-cycle variability and memory effects, significantly restricting its use in dynamic applications. Therefore, fully harnessing the potential of graphene in applications requiring dynamic device tuning demands further advancements in material synthesis, processing, and device integration. Addressing challenges such as the observed memory effect is essential for the development of reliable and efficient graphene-based components.

## Graphical abstract

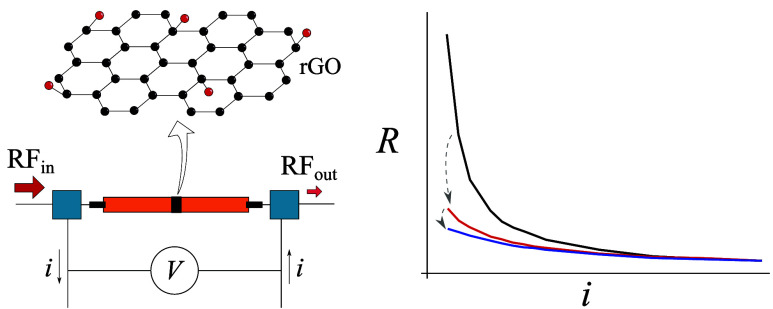

## Introduction

Due to its diverse and unique physical properties, graphene emerged as an ideal building block for next-generation devices, finding applications in several fields.^1–5^ Its mass production, however, faces challenges related to scalability, cost-effectiveness, and quality control.^6^

Graphene nanoplatelets (GnPs) present themselves as possible substitutes for monolayers in technological applications, combining low-cost, large-scale production with remarkable physical properties.^1^ Another alternative that has been explored in the past few years is graphene oxide (GO).^7,8^ Through an oxidation process, oxygen-containing functional groups may be attached to the surface of graphite, converting it to graphite oxide, and single- or few-layer GO may be produced after sonication. This system, however, presents low electrical conductivity, and further reduction processes are required to recover such a feature. The final product is known as reduced graphene oxide (rGO) and may be obtained through several distinct techniques.^7,9^

In microwave engineering, carbon-based materials are currently being tested for applications such as antennas,^10^ attenuators,^11–13^ and phase shifters.^14–16,18^ In most RF and microwave graphene-based devices, few-layer graphene is drop-casted at particular points of the circuit and functions as variable resistors, whose resistance may be tuned using a DC bias. By changing graphene’s resistance, the device can be electronically tuned. Such a feature is vital in modern communications systems with high data rates, low latency, and a massive number of users. In real scenarios, such devices must provide a reproducible response, with a long time of bias cycles. Table 1 summarizes references presenting graphene-based devices for RF applications. The type of device, the employed graphene structure, and the main results are listed.

**Table 1:** Graphene-Based Devices: Summary Table of the State-of-the-Art

article	device	graphene structure	main results
^10^	antenna	graphene-assembled film	wireless wearable RF sensor
^11^	microstrip attenuator	few-layers graphene	broadband tunable attenuator (1–20 GHz)
^12^	microstrip attenuator	few-layers graphene	broadband tunable attenuator (0–5 GHz)
			insertion loss ranging from 0.3 to 15 dB
^13^	co-planar attenuator	graphene nanoplatelets	four different working bands
			gradually covering 3.5–38 GHz
^14^	phase-shifter	few-layers graphene	phase change of 40° in
			the 5–6 GHz frequency range
^15^	phase-shifter	graphene nanoplatelets	phase change of 33°
			at 4.3 GHz
^16^	phase-shifter	graphene flakes	phase change of 59°
			at 5 GHz
^17^	microstrip attenuator	graphene nanoplatelets	broadband tunable attenuator (1–5 GHz)
^18^	substrate integrated	multilayered graphene	simultaneous amplitude and
	microstrip attenuator		phase manipulation
	and phase shifter		

The existing literature, summarized in Table 1, primarily focuses on the structural characterization of the rGO and its standard DC resistance under static bias conditions. However, to the best of the authors’ knowledge, no prior work addresses the behavior of rGO under dynamic DC bias switching. Evaluation of rGO’s ability to dynamically modulate its resistance is therefore essential for the development of graphene-based phase-shifters and attenuators for electronically tunable communication systems, such as adaptive antennas.

In this context, this paper revisits the structural characterization of rGO and its standard DC resistance analysis and further investigates its reproducibility, dynamic resistance-switching capability, and memory effects. Reproducibility is assessed through successive measurement cycles, while dynamic resistance switching and memory effects are evaluated by applying a stepwise current excitation. Four rGO samples were tested in a microstrip with a gap attenuator structure. Both DC and RF measurements were performed. All samples exhibited a variation in resistance with the applied DC bias. However, this resistance variation was not consistently reproduced in subsequent measurement cycles, resulting in a reduced dynamic variation of the graphene resistance. The results further demonstrate a resistance memory effect in rGO correlated with the magnitude of the applied current, which compromises the dynamic switching capability, along with temporal instability observed at low current levels subsequent to exposure to high current.

## Methods

Four different rGO samples were analyzed, three of them being commercially available. The fourth one, hereinafter referred to as sample S_A_, was produced by our group. GO was synthesized by the oxidation of graphite. Initially, 60 mL of concentrated sulfuric acid was added to a 500 mL round-bottom flask containing 1 g of graphite. The system was maintained under magnetic stirring for 15 min while immersed in an ice bath. After this period, 3.5 g of KMnO_4_ was gradually incorporated. The reaction mixture was stirred for an additional 2 h at room temperature. Then, 200 mL of deionized water was slowly added, and around 3 mL of 30 v/v% H_2_O_2_ solution was then introduced to complete the oxidation. The resulting graphite oxide (Gr-O) was washed with distilled water, 10% HCl solution, acetone, ethanol, and again distilled water until a neutral pH was reached. After drying, the Gr-O was dispersed in water and sonicated using a probe ultrasonicator for 10 min to promote exfoliation. The resulting GO dispersion was then reduced by refluxing with sodium borohydride (NaBH_4_) for 2 h, yielding rGO.

Sample S_B_ is sold as few-layer (2–5) rGO nanoflakes, costing 54 euros per gram. Samples S_C_ and S_D_ are marketed as rGO nanoplatelets with lateral dimensions of *l* = 1.5 and 30 μm and thicknesses of 3 and 5 nm, respectively. They are much cheaper, costing 3–6 euros per gram. Samples are listed in Table 2, where their name and specifications are indicated.

**Table 2:** Sample Identification, Lateral Dimension *l* Informed by the Seller, Flake Thickness *T*, Number of Layers *n*, and Interplanar Distance *d*_002_ Estimated by XRD

sample	specification	*l* (μm)	*T*(Å)	*n*	*d*_002_ (Å)
S_A_	homemade, 1–15 layers	0.5–5	16	4.5	3.53
S_B_	commercial, 2–5 layers	1–10	14	3.8	3.68
S_C_	commercial	1.5	154	45.6	3.38
S_D_	commercial	30	193	57.1	3.38

X-ray diffraction (XRD) measurements were performed by using a Bruker D8 Advance diffractometer in Bragg–Brentano (θ–2θ) geometry. The instrument was equipped with a copper X-ray tube and a LynxEye detector featuring silicon strip technology. The measurements utilized Cu Kα radiation (λ = 1.54 Å) under operating conditions of 40 kV and 40 mA.

Raman spectra were collected using a SENTERRA Raman microscope (Bruker) with a 532 nm laser as the excitation source. Measurements were performed in the spectral range of 1000 to 3000 cm^–1^, using a 50× objective lens. Each spectrum was acquired with 10 accumulations, and the laser power was adjusted by 50% to avoid thermal degradation of the samples. Deconvolution of the bands for the *I*_D_/*I*_G_ calculations was performed by applying a Lorentzian fitting model.

Attenuator-type devices were used in the DC and RF electrical characterization of the rGO nanoplatelets. These devices consist of two microstrip-type transmission lines, of 50 Ω each, separated by a gap where the rGO nanoplatelets are deposited. The attenuators were produced according to the design proposed in ref 17, whose schematic representation is illustrated in Figure 1, in which *W* is the width of the copper microstrips and *L* is the width of the gap between them. The attenuators were produced on a Rogers 4350B substrate with thickness *h* = 0.786 mm, dielectric constant ϵ_r_ = 3.66, and loss tangent tan δ = 0.004. The microstrips have a width *W* = 1.66 mm, which corresponds to a characteristic impedance of 50 Ω, and a length *L* = 0.830 mm, which corresponds to AR = 0.5, with the AR (aspect ratio of the gap) parameter defined as the ratio between the gap length and the width of the microstrips, AR = *L*/*W*.

**Figure 1:**
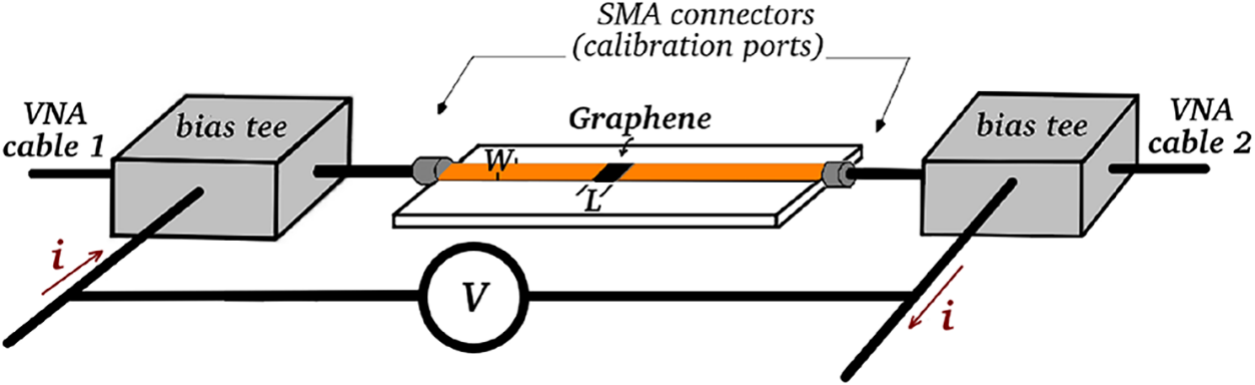
Schematic representation of the attenuators employed in RF and DC measurements.

The graphene deposition process consists of placing the attenuators on a hot plate, with the temperature adjusted to around 90 °C, and dripping an rGO/isopropanol dispersion into the gap between the microstrips. The rGO/isopropanol dispersions were prepared at a concentration of 2.0 mg/mL. To achieve this, 30 mg of rGO is mixed with 15 mL of isopropanol in a Falcon tube. The mixture is then sonicated for 5 min until a homogeneous dispersion is obtained. Using a micropipet, the dispersion is deposited dropwise into the gap of the microstripline. Since the boiling point of isopropyl alcohol is approximately 82 °C, the solvent evaporates rapidly, leaving only the rGO in the target area. The amount of rGO deposited in the gap is determined by the total volume of the dispersion applied.

To prevent the drops from spreading, mechanical containment was required. For this purpose, a square mold with a hollow interior was fabricated by using a 3D printer. Before positioning the mold on the attenuator, its lower surface, which contacts the device, is coated with silicone grease to ensure a proper seal in the deposition region. All samples were subjected to this identical process to mitigate variability in the deposition results.

In most of the tests performed, the attenuator gaps were filled with an amount of rGO ranging from 1.0 to 2.0 mg. No clear correlation between the quantity of rGO and the electrical resistance was observed; therefore, the quantities were controlled empirically. This was achieved by monitoring the electrical resistance in situ during the deposition process until the target values—typically in the range of a few kΩ—were reached.

A schematic representation of the experimental setup used for DC and RF electrical measurements can be seen in Figure 1. The attenuators, with the gap between the microstrips filled with rGO, were connected at both ends to bias-tees to ensure isolation between the DC and RF signals. For DC measurements, the device can be modeled as a variable resistor *R*_rGO_ (representing rGO) in series with a current source. As the current applied to the rGO increases, *R*_rGO_ decreases. Under DC conditions, transmission lines work as ideal conductors with negligible resistance. In the configuration shown, the DC current passes through the graphene oxide deposited in the gap between the microstrips, and the potential difference between them is measured. The resistance is obtained using Ohm’s law.

Under RF conditions, transmission lines can no longer be approximated as ideal conductors with negligible resistance, and wave propagation effects must be taken into account. In this case, the device can be modeled as two transmission lines separated by a gap that contains a thin resistive sheet. The most convenient way of evaluating RF signals is through the scattering parameters, which relate the reflected and incident waves at the device ports. For the attenuator-type device, *S*_21_ parameter represents the device transmittance between ports 1 and 2, relating the wave emerging from port 2 (reflected wave *V*_2_^–^) to the wave incident at port 1 (incident wave *V*_1_^+^). When a wave propagating from port 1 to port 2 encounters an impedance discontinuity, such as the gap containing the rGO, a portion of the wave is reflected back to port 1, while the remainder is transmitted toward port 2. Variations in rGO resistance modify the impedance mismatch in the gap, thereby affecting the reflection and transmission coefficients and, consequently, the transmittance *S*_21_.

Measurements of the transmission coefficient *S*_21_ of the tunable attenuators were made using a two-port Rohde & Schwarz ZVB-14 vector network analyzer (VNA), a pair of wideband bias tees, and a precision current source. The VNA ports are connected to the RF ends of the bias tees, which, in turn, are connected to the ends of the microstrip attenuators, as shown in the diagram in Figure 1. Using this scheme, the *S*_21_ coefficient can be measured for different frequencies between 0.1 and 14 GHZ and for different values of the DC bias current. For measurements without bias current, the resistance of the rGO electrical paths is high (hundreds of ohms), and the transmission should be low. With the application of a current of tens of mA, the resistance of the rGO decays to tens of ohms, and the signal transmission should increase considerably. For RF measurements, the calibration was performed at the SMA connectors of the test fixture, thus removing the bias tees.

The specific surface area of sample S_A_ was determined experimentally using a Quantachrome NOVA 1200 surface area analyzer. Before analysis, the sample was degassed to remove the physically adsorbed species. The BET method was applied by following standard procedures for graphene-based materials. The specific surface area values for samples S_B_, S_C_, and S_D_ were obtained from the supplier’s technical datasheets. These materials are commercially available graphene-based products, and the reported BET surface areas were adopted as reference values in this work.

## Results and Discussion

### Structural Characterization

The investigated samples exhibit distinct specific surface areas: 98 m^2^/g for S_A_, 15.62 m^2^/g for S_B_, 320 m^2^/g for S_C_, and 170 m^2^/g for S_D_, reflecting different degrees of exfoliation and stacking.

X-ray diffraction spectra obtained for the four samples are shown in Figure 2 and reveal clear differences in the structural organization of the carbon materials. Samples S_A_ and S_B_ exhibit broad and weak diffraction peaks centered at approximately 2θ ≈ 25.2° (S_A_) and 24.1° (S_B_). The interlayer spacings of the sp^2^ carbon layers, calculated using Bragg’s Law,^19,20^ are *d*_002_ ≈ 3.53 Å and 3.68 Å, respectively. These values are characteristic of partially reduced graphene oxide, reflecting the presence of structural defects, increased interlayer spacing due to residual oxygenated groups, and a lower degree of stacking order. The broadening of the (002) reflection is indicative of nanoscale crystallite domains along the stacking direction, which was quantified using the Scherrer equation.^20,21^ This technique involves using the full-width half-maximum (fwhm) of the diffraction peak—after considering the effects of experimental broadening, background, and Kα2 radiation—in the Scherrer equation to determine the average thickness of the graphene flakes (*T*).^20–23^ From this value and the interlayer spacing *d*_002_ obtained previously, the average number of graphene layers per flake can be calculated.^20^ The values of *T* and *n* obtained are presented in Table 2 and support the classification of S_A_ and S_B_ as few-layer rGO. In turn, the samples S_C_ and S_D_, marketed as graphene nanoplatelets, exhibit sharp and intense peaks centered at 2θ = 26.3°, corresponding to the (002) reflection of crystalline graphite.^24^ The narrow peak width and high intensity suggest a high degree of crystallinity and significant stacking of sp^2^-hybridized carbon layers.^25^ The calculated interlayer distances were approximately *d*_002_ = 3.38 Å, which is consistent with a well-ordered graphite. These results indicate that S_C_ and S_D_ are composed primarily of graphite nanoplatelets with tens of stacked layers rather than exfoliated graphene or reduced graphene oxide. It is important to note that while XRD provides valuable information on interlayer spacing and average stacking, it is limited in detecting amorphous or highly exfoliated regions and may underestimate the presence of single-layer or disordered sheets, especially in chemically modified graphene derivatives.^26^ In summary, the XRD data confirm that S_C_ and S_D_ are graphitic materials with high crystallinity and layer stacking, while S_A_ and S_B_ are few-layer reduced graphene oxides with greater structural disorder and expanded interlayer spacing, consistent with their synthesis history.

**Figure 2:**
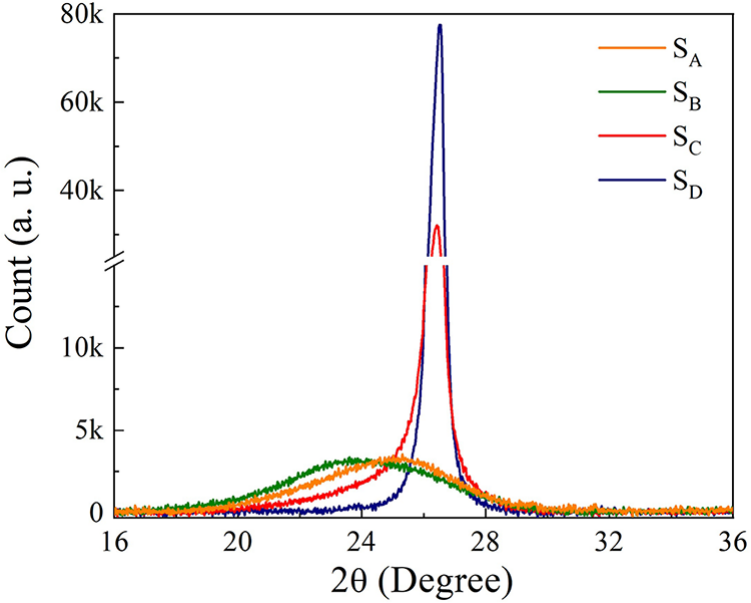
X-ray diffraction patterns are shown for the studied samples.

Raman spectroscopy was used to evaluate the degree of structural disorder and the crystalline quality of the samples. Figure 3 presents the normalized spectra obtained for the four carbon samples (S_A_, S_B_, S_C_ and S_D_), highlighting the characteristic bands: the D band (∼1350 cm^–1^), associated with breathing modes of carbon rings activated by disorder; and the G band (∼1580 cm^–1^), attributed to the intraplanar vibrations of sp^2^ hybridized carbon atoms and the 2D band (∼2700 cm^–1^), which according to its shape, intensity and width provides information about stacking and number of layers.^27–29^

**Figure 3:**
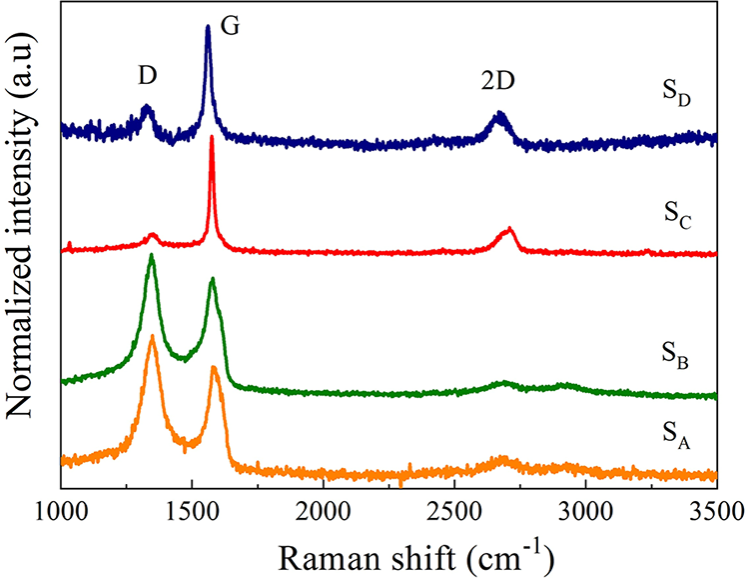
Normalized Raman spectra of samples S_A_, S_B_, S_C_, and S_D_, obtained using a 532 nm excitation laser in the spectral range of 1000 to 3000 cm^–1^.

The ratio between the intensities of the D and G bands (*I*_D_/*I*_G_) was used as a metric to quantify the degree of structural disorder. The obtained ratios were 2.6 (S_A_), 1.9 (S_B_), 0.4 (S_C_), and 0.6 (S_D_). The S_A_ sample, produced by oxidation of graphite followed by chemical reduction with sodium borohydride (NaBH_4_), presented the highest *I*_D_/*I*_G_ ratio, reflecting a high degree of disorder in the carbon network. This behavior is characteristic of rGO with highly defective structures, where the removal of oxygenated groups is not accompanied by an efficient reorganization of the sp^2^ regions.^30,31^ The prominence of the D band, combined with the low relative intensity of the G band and the discrete presence of the D′ band (∼1620 cm^–1^), confirms this high degree of structural disorganization. The 2D band, in this case, appears as a weak and broad signal, with a low *I*_2D_/*I*_G_ ratio, which reinforces the absence of electronic order and the lack of regular stacking between layers.^32,33^

Sample S_B_, a commercial rGO between 2–5 layers specification, presented *I*_D_/*I*_G_ = 1.9. Although also indicative of high disorder, the lower value compared with sample S_A_ suggests a more controlled reduction process and a partially reorganized structure. The 2D band is visible, but broad and of low intensity, without the typical profile of monolayer graphene, being compatible with rGO multilayer with some structural heterogeneity.^34^

On the other hand, the S_C_ and S_D_ samples showed significantly lower *I*_D_/*I*_G_ ratios (<1), with a clear predominance of the G band, indicating graphitic materials with low defect density. The S_C_ sample (*I*_D_/*I*_G_ = 0.4) exhibited an intense, narrow, and symmetric 2D band, with intensity comparable to that of the G band, a typical behavior of multilayer graphite nanoplatelets with ordered stacking^29,35^ (Bernal type).

The S_D_ sample (*I*_D_/*I*_G_ = 0.6) presented an equally intense 2D band, but wider and asymmetric, suggesting less regular stacking, presence of edges, structural distortions, or slight surface functionalization.^36^ This profile is consistent with that of graphite nanoplatelets. These results, combined with X-ray diffraction data, confirm that samples S_A_ and S_B_ are materials derived from rGO, with different degrees of disorder and organization, while S_C_ and S_D_ present structural features of graphite nanoplatelets, with more ordered multilayer stacking.

### DC Electrical Characterization

Figures 4(a,4c,e) shows the curves of DC electrical resistance *R* versus time *t* for three different types of rGO in three subsequent measurement cycles, named C_1_, C_2_, and C_3_. In each cycle, the measurement current *i* varied between 1 and 40 mA, and for each *i* value, *R* was measured during a time interval varying between 2 and 5 min. In all measurements, C_2_ was started immediately after the end of C_1_ and C_3_ immediately after the end of C_2_. Before the electrical measurements, all samples were in a relaxed state, in the sense that they had not been traversed by electrical current for at least 24 h prior to the beginning of the measurements.

**Figure 4:**
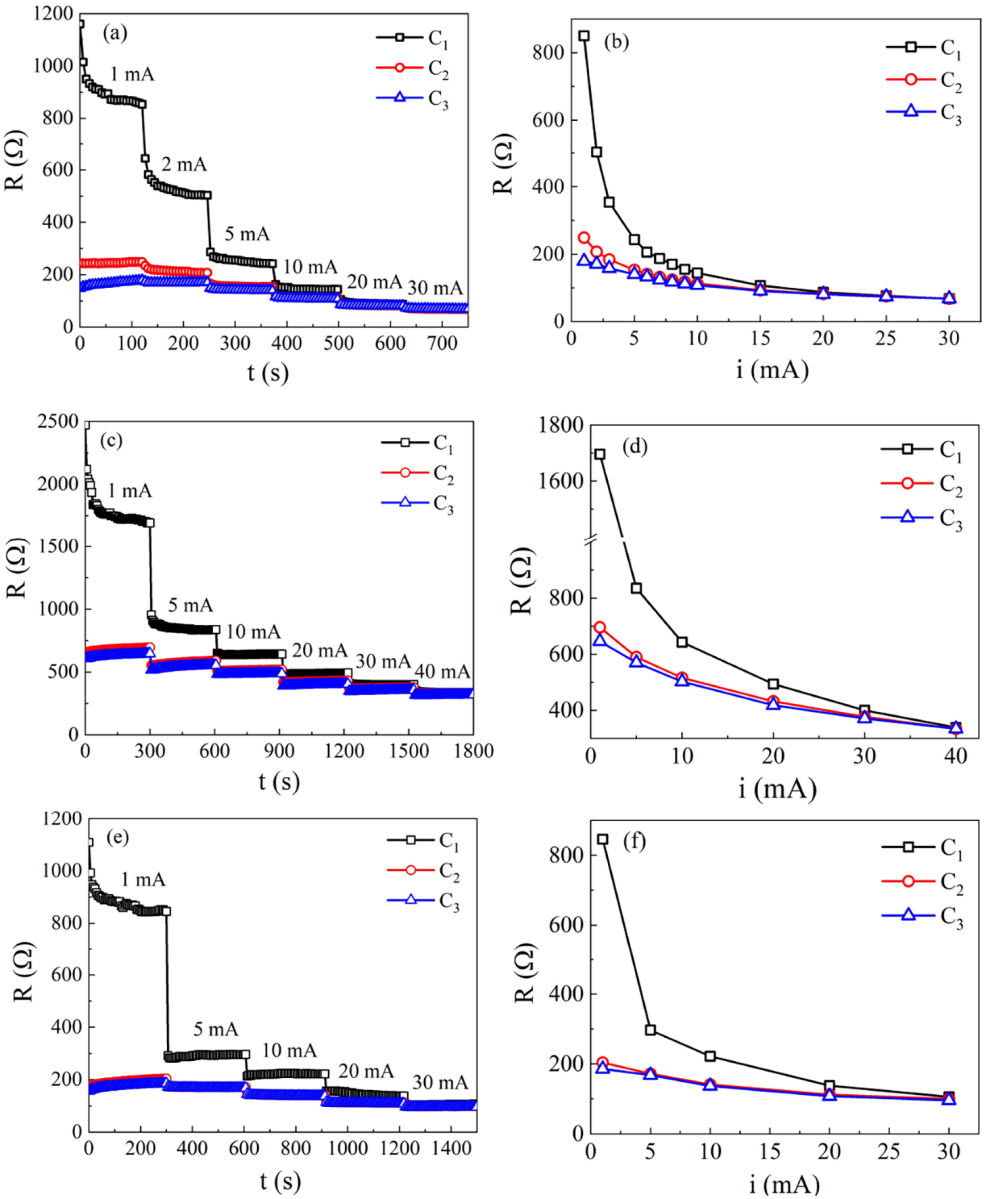
Evolution of the attenuator’s electrical resistance with the applied current. Panels (a) and (b) display results obtained for sample S_A_, (c) and (d) for S_B1_, and (e) and (f) for S_D_.

The *R*(*t*) curves in Figure 4(a) were obtained with a sample of approximately 2 mg of rGO-type S_A_ (produced at the UFSM). It can be observed that, at the beginning of cycle 1 (black symbols), when *i* = 1 mA, *R* decays with time from ∼1.2 kΩ to ∼850 Ω; when the current increases to *i* = 2 mA, *R* drops abruptly to ∼650 Ω, decreasing with time to ∼500 Ω. The electrical resistance continues to decrease as *i* increases, reaching a minimum value of ∼75 Ω when *i* = 30 mA, the highest current value applied in the cycle. It is also possible to observe that the temporal variation of *R*, for a fixed value of *i*, decreases for currents above 5 mA. Thus, at the end of C_1_, the value of *R* decreased from ∼1.2 kΩ to ∼75 Ω, a result similar to those described in the literature.^12–16^ However, what is not described in the literature is how *R* responds if a new measurement cycle is triggered immediately after the end of C_1_. The curve for C_2_ (red symbols) shows that when *i* is adjusted to 1 mA immediately after the end of C_1_, the value of *R* does not return to C_1_’s initial value of ∼1.2 kΩ, but to a much lower value of ∼250 Ω. As in C_1_, the value of *R* decreases with the increase of *i*, reaching a minimum value of ∼75 Ω when *i* = 30 mA at the end of C_2_. Thus, in C_2_, the value of *R* decreases from ∼250 Ω to ∼75 Ω, a variation much lower than that obtained in the first cycle. The curve for C_3_ (blue symbols) shows that, at the beginning of this cycle, when *i* = 1 mA, the initial value of *R* is ∼150 Ω, a value even lower than the initial value of *R* in C_2_, increasing over time to ∼200 Ω. As in the previous cycles, *R* decreases as *i* increases, reaching a minimum value of 75 Ω for *i* = 30 mA. Thus, in C_3_, the value of *R* decays from 200 Ω to 75 Ω, an even lower variation than that observed in C_2_.

The *R*(*i*) curves in Figure 4(b) summarize the results described above: the *R* values used in these graphs were extracted from the graphs in Figure 4(a) and correspond to the values obtained from the last measurement of each 2 min interval for each applied current. It is clear that the large decay of *R* with an increasing current observed in C_1_ is not repeated in C_2_ and C_3_. Note that for current values below 5 mA, the *R* values show significant differences between measurement cycles. However, for currents above 5 mA, the C_2_ and C_3_ curves are nearly identical. Additionally, for currents above 5 mA, all three cycles, C_1_, C_2_, and C_3_, exhibit very similar resistance values.

Figure 4(c) shows the *R*(*t*) curves for three measurement cycles obtained with a 2 mg sample of rGO-type *S*_B_ (high-quality commercial, 3–5 layers). This sample will be referred to as *S*_B1_. The electrical response of this sample is qualitatively very similar to the response presented by sample S_A_. The *R*(*i*) curves in Figure 4(d) summarize the results obtained with the *S*_B1_ sample. Again, the large decay of *R* with the increase of *i* observed in C_1_ is not repeated in C_2_ and C_3_. On the other hand, the curves of C_2_ and C_3_ practically overlap, with a more pronounced discrepancy only for *i* = 1 mA. In C_3_, *R* decays from ∼650 Ω (*i* = 1 mA) to ∼320 Ω (*i* = 40 mA), a variation of ∼330 Ω, which is greater than the variation of ∼125 Ω observed for sample S_A_ in C_3_. Furthermore, this sample proved to be more resistant than that of S_A_.

Figure 4(e,f) shows the *R*(*t*) and *R*(*i*) curves for three measurement cycles obtained with a 2 mg type S_D_ rGO sample (commercial, cheap, with *l* = 30 μm). The results are numerically very similar to those obtained for sample S_A_. Again, the large decrease in *R* with *i* observed in C_1_ is not repeated in C_2_ and C_3_. The curves of C_2_ and C_3_ are practically superimposed.

For all rGO samples, the resistance values in the first measurement cycle differed significantly from those in subsequent cycles at the same current. However, starting from the second cycle, the *R* versus *i* curves nearly overlap, particularly for current values exceeding 5 mA.

Figure 5(a,b) shows *R*(*t*) curves for different current values, obtained with a sample of 2 mg of rGO-type S_C_ (commercial, cheap, *l* = 1.5 μm) and another sample of 1 mg of rGO-type S_B_ (commercial, expensive, 3–5 layers), respectively. The latter will be referred to as *S*_*B*2_. In both cases, the applied current was adjusted in steps, ranging from 0.1 to 80 mA. However, after each measurement performed with a current *i* ≥ 10 mA, a new temporal measurement was conducted at *i* = 1 mA. The intention here was to test the dynamic switching capability of *R*. For each applied current, *R* was measured over a period of 3 min. Before the measurements, both samples were in a relaxed state, as defined previously.

**Figure 5:**
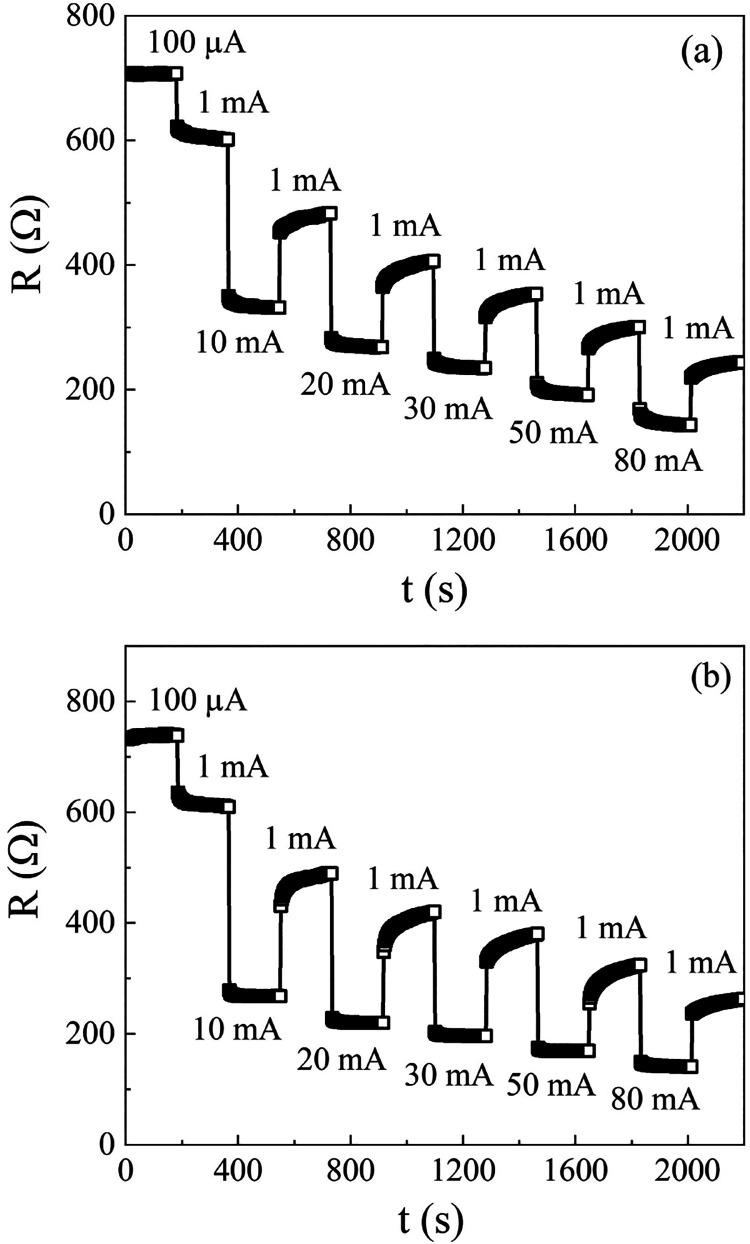
Time evolution of *R* as the DC current is switched between 1 mA and an increasing value of *i*, ranging from 0.1 to 80 mA. Results were obtained for (a) S_C_ and (b) S_B2_.

The graphs in Figure 5 show that, for both samples, *R* decays with the applied current, varying from an initial value of ∼700 Ω, when *i* = 0.1 mA, to ∼140 Ω, when *i* = 80 mA, representing a variation of 560 Ω. However, it also shows that the resistance *R*(1 mA) is dependent on the previously applied current. For example, when *i* = 1 mA is applied after *i* = 100 μA, *R*(1 mA) is approximately 600 Ohms. In contrast, when *i* = 1 mA is applied after *i* = 80 mA, *R*(1 mA) drops to around 250 Ohms. This behavior results in a decrease in the dynamic variation of resistance. The results in Figure 5 also show that after applying a current *i* > 10 mA, the response of *R* at *i* = 1 mA is not stable but instead increases over time. The memory effect of *R* with respect to the previously applied current, along with its temporal instability at low current following the application of a high current, presents a challenge for the use of graphene oxide in electronically controlled devices, especially those that rely on dynamic resistance switching for electronic tuning.

The structural disorder identified by XRD and Raman analysis reveals important differences in the microstructures of the studied samples. Namely, S_A_ and S_B_, which feature broad (002) reflections and elevated *I*_D_/*I*_G_ ratios, possess a flawed rGO network marked by numerous oxygenated sites and disrupted sp^2^ domains. These characteristics lead to unstable percolation routes and charge-trapping centers, which could account for the significant decrease in resistance during the initial current cycle and the noticeable memory effect in subsequent cycles. On the other hand, S_C_ and S_D_ exhibit sharp, graphite-like diffraction peaks and low *I*_D_/*I*_G_ ratios, indicating well-organized multilayer stacking. However, as can be seen in Figures 4 and 5, all of the samples present very similar electrical responses. This suggests that the observed memory effect may be connected to interflake processes and not only to intraflake ones.

The specific surface area values of the investigated samples further support this interpretation. The BET surface areas span more than 1 order of magnitude, ranging from 15.62 m^2^/g for S_B_ to 320 m^2^/g for S_C_, with intermediate values of 98 m^2^/g for S_A_ and 170 m^2^/g for S_D_. In these quasi-two-dimensional materials, the accessible surface area is primarily determined by the degree of exfoliation, stacking, and the density of interfacial junctions rather than by intrinsic porosity. Therefore, samples with a higher surface area are expected to exhibit more interflake contacts and interface-dominated conduction pathways, which enhance charge trapping and current-induced rearrangements at junctions, thereby contributing to the observed cycle-to-cycle variability and memory effects. Conversely, the much lower surface area of S_B_ suggests a more compact stacking with fewer accessible junctions, consistent with a reduced contribution of interface-driven effects. These results reinforce that interflake processes predominantly govern the memory behavior observed in this work.

### RF Electrical Characterization

Figure 6 presents the RF measurements of samples S_A_, S_B1_, and S_D_, whose DC characterizations are shown in Figure 4. The plots display |*S*_21_| as a function of frequency for zero current and for the maximum current applied to each sample, as defined in Figure 4. All measurements were performed at a power level of *P* = 0 dBm. The curves corresponding to *i* = 0 (black symbols) were obtained with the samples in a relaxed state; therefore, the *R*(*i* = 0) values are higher than the initial values shown in Figure 4(a,c,d). The maximum current values are the same as those used in the DC measurements; consequently, the resistance values under maximum current application correspond to the minimum resistance shown in Figure 4(b,d,e).

**Figure 6:**
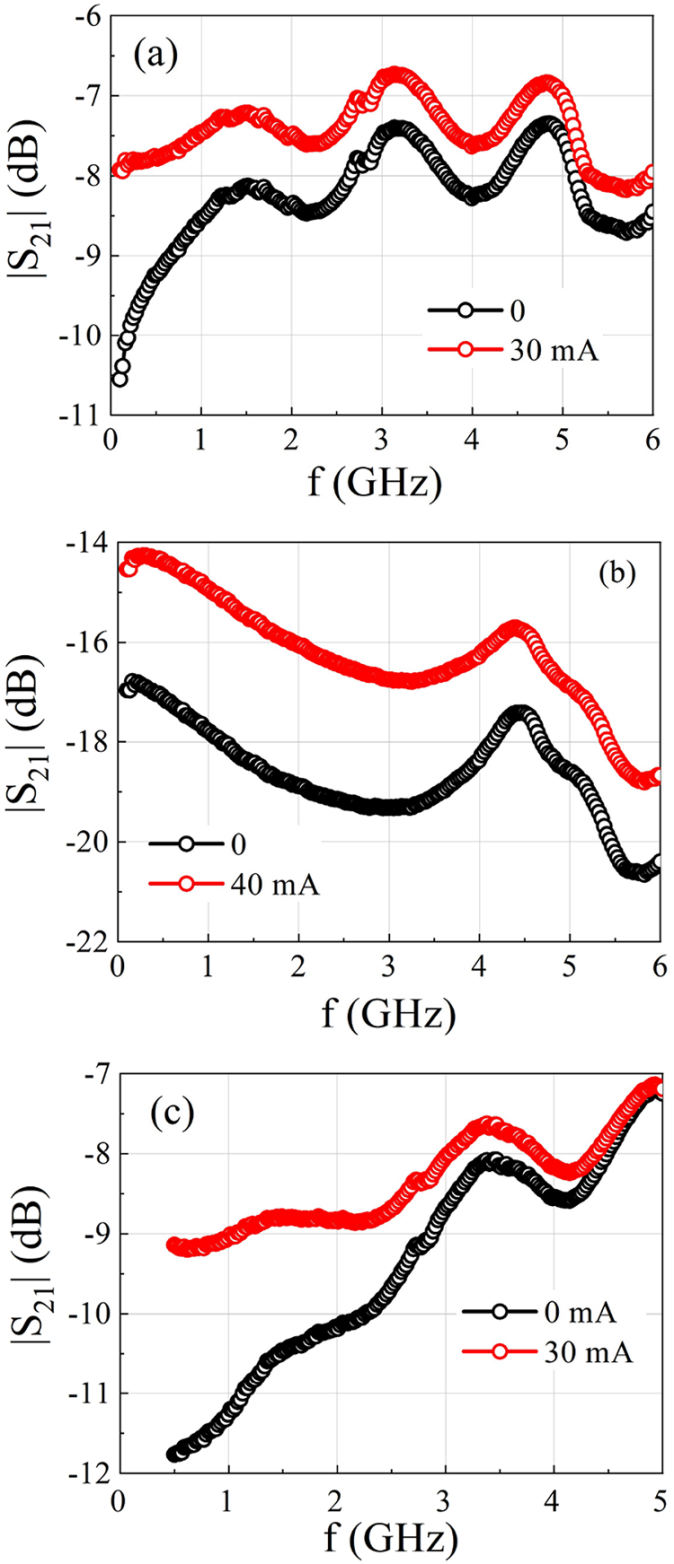
Transmittance obtained using two different DC currents for (a) S_A_, (b) S_B1_, and (c) S_D_.

The curves in Figure 6 show that, for all samples, the transmittance increases with the application of the bias current, as expected due to the decrease in graphene resistance *R*. The results indicate that samples S_A_ and S_D_ exhibit more pronounced shifts in the transmittance curves at lower frequencies. Sample S_A_, for example, shows a shift of approximately 2.5 dB between the curves measured at 0 and 30 mA at 100 MHz, whereas this shift decreases to about 1 dB at 1 GHz. A similar trend is observed for sample S_D_. In contrast, sample S_B1_ (high-quality commercial rGO, 3–5 layers) demonstrates superior performance, maintaining a consistent shift of approximately 2 dB between the curves throughout the analyzed frequency range.

Figure 7 shows the transmittance as a function of frequency for selected current steps applied to samples S_C_ and S_B2_, corresponding to the DC results presented in Figure 5. Solid lines represent positive current steps (i.e., an increase in the applied current), while dashed lines represent negative current steps (i.e., a decrease in the current). The two values shown in each legend indicate the values of the current step. For example, 1mA_10mA denotes an increase from 1 to 10 mA, whereas 80mA_1mA indicates a decrease from 80 to 1 mA. These results confirm the memory effect observed in the DC characterization. Notably, in both samples, when the current is reduced from 80 mA to 1 mA, the transmittance curve does not return to the same position as that observed for the step from 100 μA to 1 mA. In the same figure, the black lines represent curves obtained for 100 μA for relaxed samples.

**Figure 7:**
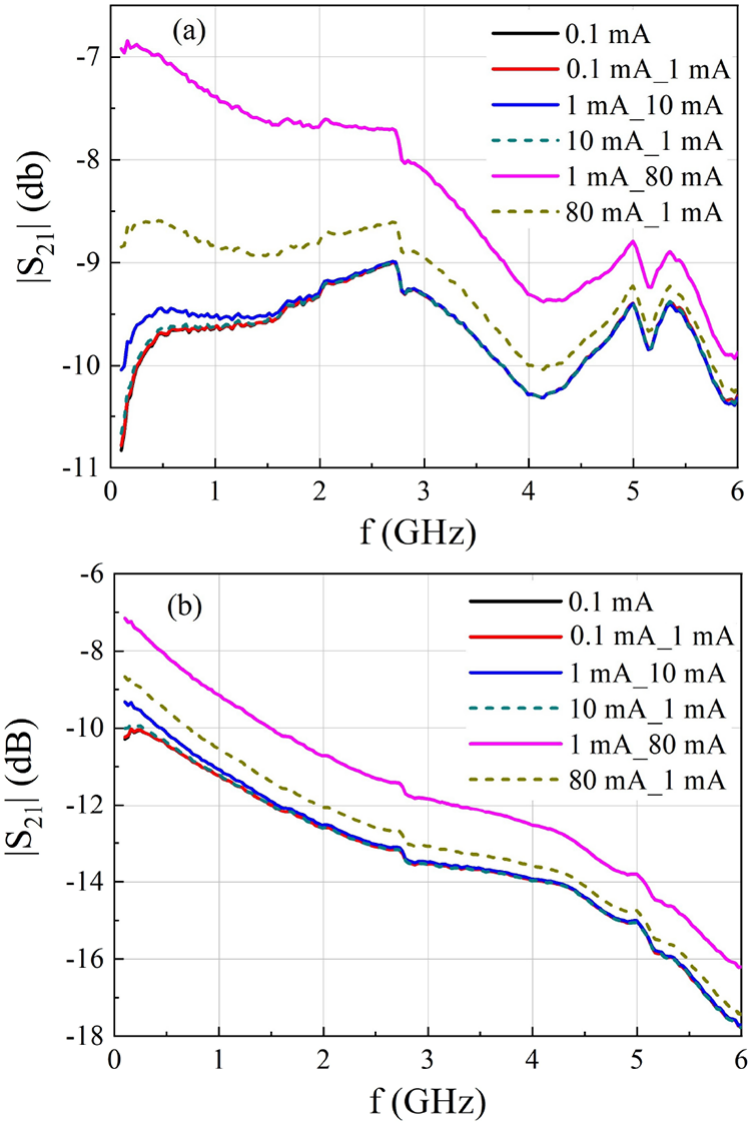
Transmittance as a function of frequency for selected current steps applied to samples (a) S_C_ and (b) S_B2_.

## Conclusions

This paper presents an investigation of the measurement cycle reproducibility and dynamic resistance-switching behavior of reduced graphene oxide and graphite nanoplatelets. Structural characterization and DC and RF analyses were performed on four graphene-based samples.

The structural analysis revealed that two of the samples were few-layer rGO, exhibiting different degrees of disorder and structural organization, while the other two were identified as graphite nanoplatelets, characterized by more ordered multilayer stacking.

DC electrical measurements demonstrated that graphene resistance can be controlled via a DC current, ranging from an initial resistance of several kilo-ohms to values as low as a few tens of ohms. However, all samples exhibited a memory effect in their electrical resistance, which was dependent on the magnitude of the applied DC current. This memory effect was evaluated over three measurement cycles. At the end of the first cycle, when the DC current was reduced to near zero, the resistance did not return to its initial value but settled at a significantly lower level. The same behavior was observed between the second and third cycles. This effect results in a substantial reduction in the dynamic resistance variation for a given range of applied currents, particularly between the first and second cycles. Moreover, as the applied current increases, the resistance tends to converge to similar values across all measurement cycles. Further results revealed that the high resistance values observed at near-zero currents are dependent on the magnitude of the previously applied current, and their value increases over time.

RF measurements showed that the sample transmittance is highly dependent on the sample characteristics. The evaluated samples presented maximum transmittance variation (for the applied range of current) of 4 dB, which is significantly lower than the values previously reported in the literature.^11,17^ The results presented in this paper demonstrate that it is possible to control the resistance of graphene; however, they also highlight the material’s memory effects across different measurement cycles, the dependence of high resistance values (at low current) on the previously applied current, and its temporal instability, phenomena not addressed in the existing literature.

Graphene-based RF and microwave devices hold significant promise as key components in future high-performance technologies due to their exceptional electrical and mechanical properties. However, realizing the full potential of graphene in these applications requires continued advancements in materials synthesis, processing techniques, and device integration. Addressing the current challenges, such as the memory effect reported in this paper, is essential to enable reliable and efficient graphene-based components. Therefore, sustained research and development efforts are imperative to overcome these obstacles and pave the way for widespread adoption of graphene in RF and microwave device technologies.
